# Modeling Physiological Sources of Heading Bias from Optic Flow

**DOI:** 10.1523/ENEURO.0307-21.2021

**Published:** 2021-11-10

**Authors:** Sinan Yumurtaci, Oliver W. Layton

**Affiliations:** Department of Computer Science, Colby College, Waterville, ME 04901

**Keywords:** heading, model, motion, MSTd, MT, optic flow

## Abstract

Human heading perception from optic flow is accurate for directions close to the straight-ahead and systematic biases emerge in the periphery (Cuturi and Macneilage, 2013; Sun et al., 2020). In pursuit of the underlying neural mechanisms, primate brain dorsal medial superior temporal (MSTd) area has been a focus because of its causal link with heading perception (Gu et al., 2012). Computational models generally explain heading sensitivity in individual MSTd neurons as a feedforward integration of motion signals from medial temporal (MT) area that resemble full-field optic flow patterns consistent with the preferred heading direction (Britten, 2008; Mineault et al., 2012). In the present simulation study, we quantified within the structure of this feedforward model how physiological properties of MT and MSTd shape heading signals. We found that known physiological tuning characteristics generally supported the accuracy of heading estimation, but not always. A weak-to-moderate overrepresentation of peripheral headings in MSTd garnered the highest accuracy and precision out of the models that we tested. The model also performed well when noise corrupted high proportions of the optic flow vectors. Such a peripheral MSTd model performed well when units possessed a range of receptive field (RF) sizes and were strongly direction tuned. Physiological biases in MT direction tuning toward the radial direction also supported heading estimation, but the tendency for MT preferred speed and RF size to scale with eccentricity did not. Our findings help elucidate the extent to which different physiological tuning properties influence the accuracy and precision of neural heading signals.

## Significance Statement

Using vision to perceive direction of self-motion (heading) lies at the heart of our ability to effectively move through the world. Prior work has shown that monkey brain dorsal medial superior temporal (MSTd) area is involved in heading perception. We simulated in a computational model how physiological tuning properties of areas medial temporal (MT) and MSTd influence neural heading signals. We found that a neural representation of heading biased toward the periphery, in combination with other factors, best supported the accuracy and precision of heading estimates. We draw on existing models and known physiology to promote the broad applicability of our findings. Our analysis helps improve our understanding of the neural mechanisms underlying heading perception.

## Introduction

**Figure 1.**
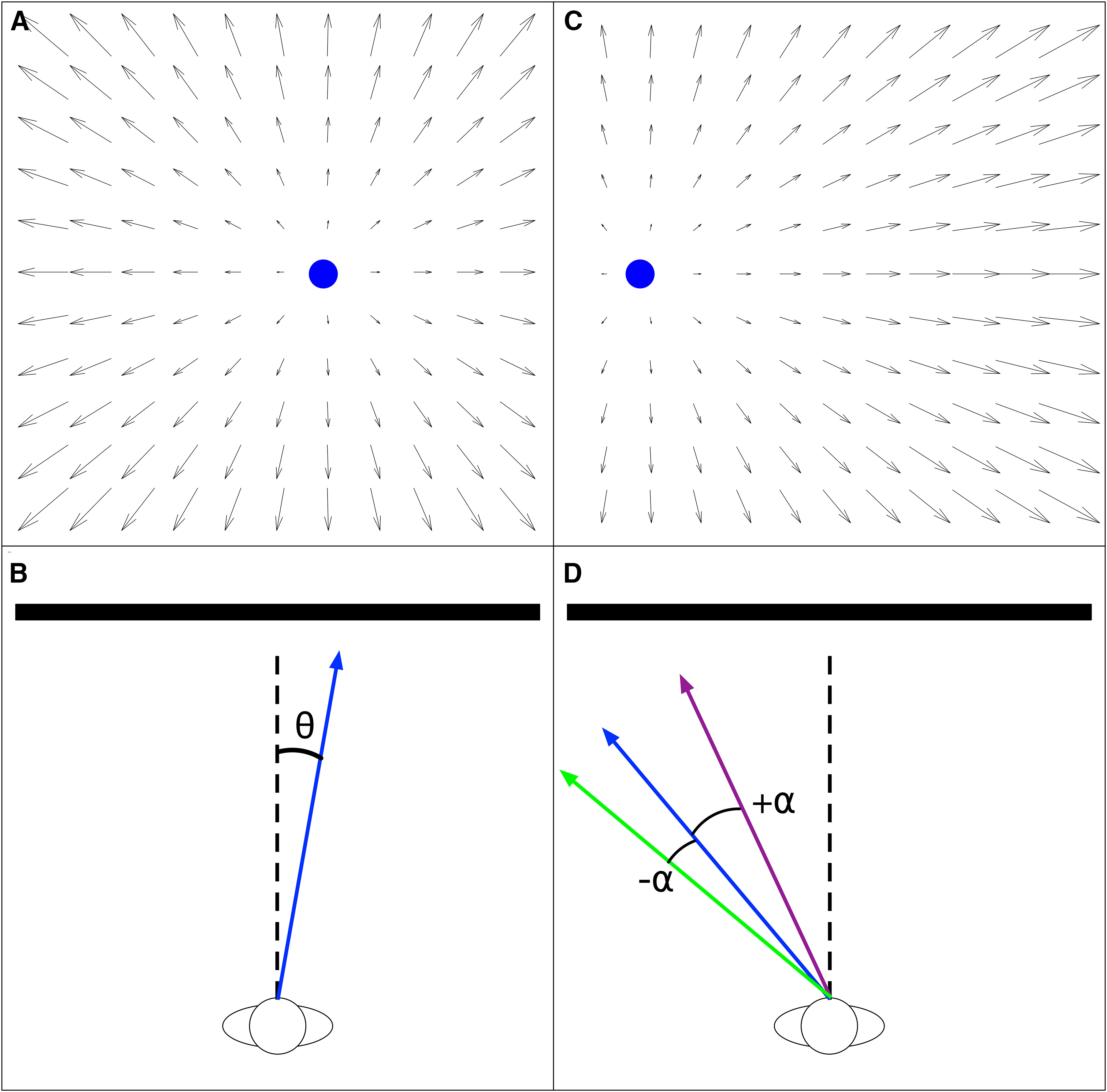
***A***, Idealized optic flow experienced by an observer heading +10° to the right of the straight-ahead direction (FoE indicated by blue disk). ***B***, Bird’s eye view of scenario in ***A***. Dashed line indicates the straight-ahead direction, blue solid arrow indicates the observer’s heading *θ*. ***C***, Optic flow experienced by an observer moving at a peripheral −40° heading. ***D***, Top-down bird’s eye view of the scenario from ***C***, which highlights our convention for representing heading bias. Purple solid arrow illustrates underestimation of the judged heading angle or center bias (heading error *α* > 0° toward the straight-ahead direction) and green solid arrow illustrates overestimation of the judged heading angle or peripheral bias (heading error *α* < 0° away from the straight-ahead direction). The optic flow shown in top panels reflects a 90° field of view.

Self-motion through the world produces expansive motion patterns on the eye known as optic flow (Gibson, 1950). The optic flow radiates from a single point known as the focus of expansion (FoE) that specifies the direction of travel (heading) when the observer moves forward along a straight path through a rigid environment without sources of rotation, such as eye movements (Fig. 1*A*,*B*). Under such conditions, humans are indeed capable of accurately perceiving heading from optic flow (Warren and Hannon, 1988; Van Den Berg, 1992), although errors in heading judgments do arise when the FoE is offset from the straight-ahead direction (i.e., observer moves toward the periphery; Fig. 1*C*,*D*; Warren and Kurtz, 1992; Crane, 2012; Cuturi and Macneilage, 2013). The error tends to be biased toward the center (straight-ahead direction) and ranges from several degrees for central headings to 10° at the periphery (Fig. 1*D*, *α* > 0; Llewellyn, 1971; Johnston et al., 1973; Cutting et al., 1992; Warren and Saunders, 1995; Royden and Hildreth, 1996; Layton and Fajen, 2016b; Sun et al., 2020).

While the neural basis of heading perception is unknown, a causal link has been established with the dorsal medial superior temporal area (MSTd) in primate cortex (Britten and Van Wezel, 1998; Gu et al., 2012). MSTd neurons demonstrate sensitivity to full-field motion patterns that resemble those experienced during self-motion (Fig. 1*A*,*C*; Duffy and Wurtz, 1995; Gu et al., 2006). Tuning to the FoE position in the pattern along with numerous other visual and nonvisual signals that arise during self-motion has supported the view that MSTd produces neural signals that reflect heading estimation (Tanaka et al., 1986; Duffy and Wurtz, 1991; Graziano et al., 1994; Gu et al., 2006). An important predictor of MSTd activity is the medial temporal area (MT; Mineault et al., 2012), a motion sensitive area situated earlier in the visual hierarchy (Van Essen and Maunsell, 1983) that sends feedforward signals to MSTd. Most motion-sensitive neurons in MT are tuned to direction and speed and have much smaller receptive fields (RFs) than neurons in MSTd (Born and Bradley, 2005).

**Figure 2.**
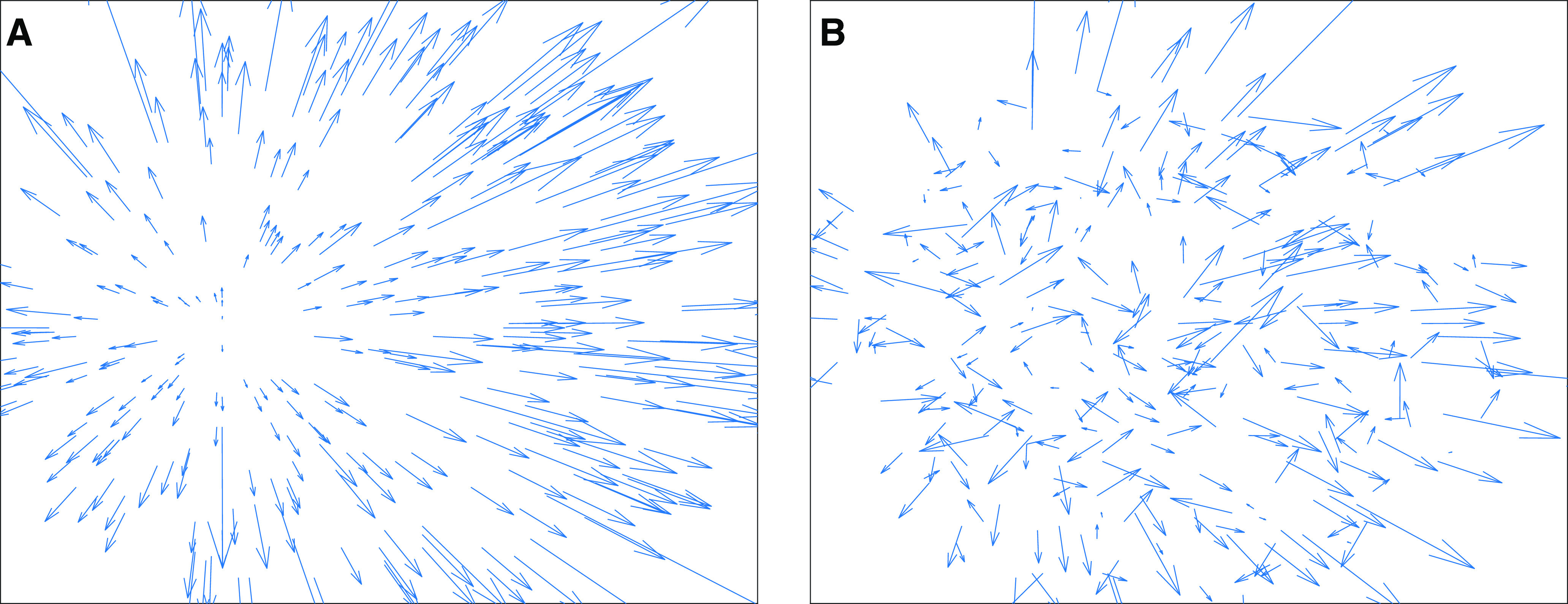
Sample frames from optic flow stimuli. ***A***, Self-motion along a −20° heading through a 3D dot cloud. ***B***, Same scenario as ***A*** with 70% noise (*n *=* *0.7).

The prevailing hypothesis is that heading sensitivity emerges in MSTd through the feedforward integration of local, MT-like motion signals across the visual field in specific radial motion “templates” that are compatible with the preferred heading direction (Fig. 2*A*). This view has been supported by computational models, which have helped elucidate possible neural mechanisms and make quantitative predictions (Perrone and Stone, 1998; Royden, 2002; Browning et al., 2009; Layton et al., 2012; Perrone, 2012). Despite their focus on biological plausibility, these models tend to implicitly assume that MT and MSTd cells possess uniform sensitivity to heading, direction, speed, and other physiological properties across the visual field. This assumption, which has not been supported by neural data (Born and Bradley, 2005; Britten, 2008), may have important consequences in our understanding of the neural mechanisms underlying heading perception.

Our aim was to investigate how known physiological tuning properties (e.g., heading, direction, and speed; RF size) may interact to shape heading signals. We explored the influence of neural tuning in a template model of MT-MSTd focused on simplicity to make our findings broadly applicable. Our simulations quantify how the following physiological properties of MT and MSTd influence heading estimation:

- The degree to which tuning across MSTd overrepresents central or peripheral headings (Gu et al., 2006).
- The considerable range of MSTd RF sizes (Tanaka et al., 1986).
- Bias in MT direction tuning toward a global radially expanding pattern (Albright, 1989).
- The tendency for MT preferred speed (Maunsell and Van Essen, 1983a; Mikami et al., 1986) and RF size (Tanaka et al., 1986; Raiguel et al., 1995) to increase with eccentricity.

Our simulations characterize the accuracy and variability of heading estimates derived from families of MT and MSTd models that implement different physiological tuning properties. To contextualize the results, we considered both models constrained by findings from the aforementioned physiological studies and counterfactual models. For example, we simulated MSTd models all along the center-peripheral continuum, not only those with an overrepresentation of peripheral headings (Gu et al., 2006). Our second major goal was to characterize the sensitivity of each model under non-idealized conditions. To this end, we simulated each model with optic flow that contains noise in the direction and speed of the motion vectors.

## Materials and Methods

### Optic flow stimuli

Optic flow produced by a virtual observer translating through a 3D dot cloud served as the input to the neural models of MT and MSTd. We represented the environment in a standard 3D coordinate system wherein the observer’s eye was centered at the origin, the positive *z*-axis aligned with the optical axis in front of the observer, and the *x*- and *y*-axes followed right-handed conventions. The scene contained 300 dots (*T*) that occupied random 3D (X,Y,Z) positions within a 300 × 300 × 100 m volume beginning 1 m front of the observer (Table 1). Each model simulation consisted of a two second, 60 frame (*F*) digital video of simulated self-motion at a speed of 1.5 m/s (*o*) along one of 21 heading directions (*θ*) equally spaced along the horizontal midline (i.e., *x*-axis): between −50° and +50° in steps of 5°. We generated the corresponding optic flow analytically using the optic flow equations (Longuet-Higgins and Prazdny, 1980):

(1) $$\left(\begin{array}{c} \dot{x}\\ \dot{y} \end{array}\right)=\frac{1}{Z}\left(\begin{array}{ccc} -f & 0 & x\\ 0 & -f & y \end{array}\right)\left(\begin{array}{c} T_{x}\\ T_{y}\\ T_{z} \end{array}\right),$$

where $\dot{x}$ and $\dot{y}$ represent the horizontal and vertical components, respectively, of the motion vector that occupies position (x,y) on the simulated observer’s retina, *f* is the focal length of the model eye and the vector $\vec{T}=\left(T_{x},T_{y},T_{z}\right)\prime$ represents the observer’s instantaneous translation through the environment. We derived the 2D retinal coordinates (x,y) by projecting the 3D dots using the standard pinhole camera model: (x,y)=f/Z(X,Y). We scaled and discretized the positions and motion vectors $\left(x,y,\dot{x},\dot{y}\right)$ to a 128 × 128 pixel grid ( $\vec{p}$). By simple trigonometry and the fact that the observer only moved along horizontal headings, the translation vector reduces to $\vec{T}=\left(\mathrm{o*sin}\theta ,0,\mathrm{o*cos}\theta \right)\prime$.

**Table 1:** Parameters that characterize the optic flow inputs used to simulate neural models of MT and MSTd

Parameter	Description	Value
$\vec{e}$	3D dot cloud extent	300 × 300 × 100 m
θ	Observer heading	$\left[-50^{{}^{\circ}},-45^{{}^{\circ}},...,50^{{}^{\circ}}\right]$
*o*	Observer speed	1.5 m/s
*h*	Observer height	1.61 m
*v*	Field of view	90°
f	Focal length	1.74 cm
*T*	Dots in scene	300
*n*	Noise dot proportion	[0.7,0.8,0.9]
*n_m_*	Maximum random (*X*, *Y*, *Z*) displacement of each noise dot around its constant observer-relative position on each frame	1 m
*F*	Duration	2 s (60 frames)
$\vec{p}$	Spatial resolution	128 × 128 pixels

We maintained a constant optic flow density throughout each video by clipping and replacing motion vectors that exited the 90° field of view (v) or came within 1 m in depth of the observer. Figure 2*A* shows a sample frame of optic flow corresponding to self-motion along a −20° heading. Table 1 summarizes the parameters used to generate the optic flow inputs.

### Noise conditions

We designed noise conditions that manipulated the signal-to-noise ratio in the optic flow field, emulating the approach of Van Den Berg (1992). We replaced a proportion *n* of the 300 dots in the 3D cloud with those that moved randomly in a reference frame that moved with the observer. These “noise dots” did not appear to approach the observer throughout each video like the rigid “non-noise dots” did. Instead, they occupied a constant mean position in the 3D dot cloud relative to the observer and drifted by a random amount between 0 and *n_m_* independently in *X*, *Y*, and *Z* on each frame of video. As with the optic flow stimuli without noise, we maintained a constant dot density across the display by replacing dots that leave the field of view. This type of noise had the desirable effect of introducing locally discrepant directions across the optic flow field with variations in speed that both were anchored in the background statistics and did not drastically change throughout the video. Figure 2*B* shows a sample frame of optic flow corresponding to self-motion along a −20° heading with noise level *n *=* *0.7. We simulated three noise levels: 70%, 80%, and 90%. We selected such high proportions of noise following preliminary testing that showed that most model parameters were robust to more moderate amounts of noise.

### Neural model of MT and MSTd

**Figure 3.**
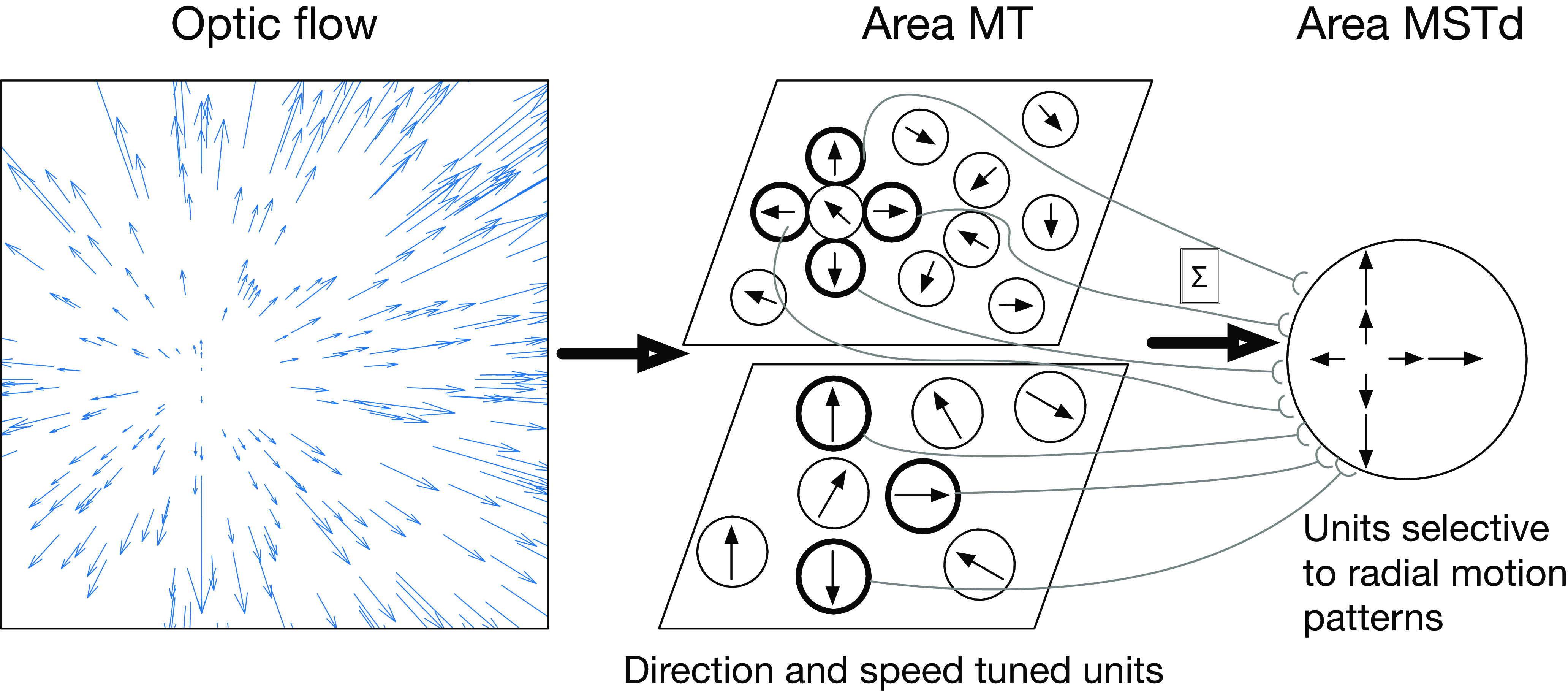
Schematic showing the architecture of MT-MSTd template models. Direction and speed tuned units in MT activate to the optic flow input and send feedforward signals to MSTd. Connections are structured such that tuning preferences in MT match a radial template pattern consistent with self-motion in a particular direction.

We simulated a neural model of brain areas MT and MSTd that has the same fundamental structure as existing template models (Hatsopoulos and Warren, 1991; Warren and Saunders, 1995; Perrone and Stone, 1998; Royden, 2002; Browning et al., 2009; Raudies and Neumann, 2010; Layton et al., 2012; Perrone, 2012). Our aim was not to implement any specific model; rather we sought to create a “default” template model that encapsulates computations that are fundamental to the family. In a series of computational experiments, we either substituted mechanisms or varied model parameters to assess the influence on heading estimates. To focus on the computations within MT and MSTd, we adhered to an assumption commonly made by template models that the optic flow input directly stimulates model MT units, abstracting away V1 and other earlier areas (Fig. 3).

We begin by presenting the default MT-MSTd template model structure and its parameters. Subsequent sections describe which specific parametric and mechanistic variations that were chosen to construct different families of the template model. We selected the default structure and parameter values by examining which produced the most accurate heading estimates within physiologically plausible ranges, all other factors remaining equal. Note that because this selection process considered the impact of each parameter on heading estimates one at a time, the resulting set of parameters may not correspond to the global optimum in the model’s large parameter space. Table 2 summarizes the default parameters used in model MT simulations and Table 3 summarizes those in model MSTd.

**Table 2:** Summary of default model MT parameters used in simulations

Parameter	Description	Value
*N_MT_*	Number of MT units	225
$\vec{c}=\left(c_{x},c_{y}\right)$	Coordinates of visual field left	$\left(45^{{}^{\circ}},45^{{}^{\circ}}\right)$/ (64,64) pixels
$\vec{r}=\left(r_{x},r_{y}\right)$	Coordinates of MT RF left	Arranged in a 15 × 15 grid spaced apart by 7.1° (8 pixels)
*σ_d_*	Maximum extent of random deviation in MT direction preference about the radial direction	180°
*σ_r_*	Effective RF radius	6° (7 pixels)
*σ_v_*	Sensitivity to optic flow that differs from the preferred direction	10°
*σ_s_*	Sensitivity to optic flow that differs from the preferred speed	0.5°/s (0.45 pixels/s)
*α_MT_*	MT activation passive decay rate	0.1
*β_MT_*	MT activation upper bound	2.5
*dt*	Integration time step	0.1 ${\text{frame}}^{}\left(0.003{\text{ sec}}^{}\right)$

**Table 3:** Summary of default model MSTd parameters used in simulations

Parameter	Description	Value
*N_MST_*	Number of MSTd units	169
γ	Controls left-peripheral bias in heading representation	0.5 (“peripheral model”), 2 (“central model”)
*σ_MST_*	Controls size of MSTd RFs	77 pixels $\left(50^{{}^{\circ}}\right)$
*α_MST_*	MSTd activation passive decay rate	0.1
*β_MST_*	MSTd activation upper bound	2.5

### Model area MT

#### MT unit placement

Model area MT consists of 225 (*N_MT_*) direction and speed tuned units that integrate optic flow within their RF (Fig. 3). In experiments focusing on MSTd, we arranged the RF centers in a 15 × 15 grid spaced by 7.1° (measured relative to the fovea) to afford overlap among adjacent RFs. We otherwise distributed their RFs randomly across visuotopic space.

#### MT direction selectivity

We parameterized MT direction selectivity around the physiological bias toward an idealized radial pattern that radiates out from the center of the visual field (0° heading; Albright, 1989). Each MT unit’s direction preference obeys

(2) $$d^{*}=\arctan\left(\frac{r_{y}-c_{y}}{r_{x}-c_{x}}\right)+x_{d},$$

where $\vec{c}=\left(c_{x},c_{y}\right)$ indicates the center position of the visual field and $\vec{r}=\left(r_{x},r_{y}\right)$ indicates the position of the RF center. The variable *x_d_* is a random variate sampled from the uniform distribution

(3) $$x_{d}∼U\left[-\sigma_{d}/2,\sigma_{d}/2\right],$$

where *σ_d_* is the two-sided maximum extent of random deviation. Taken together, Equations 2, 3 assign each cell a random direction preference centered on the idealized radial direction based on the MT cell’s RF position. In the default model configuration, we set *σ_d_* = 180°, which means that each MT unit’s direction preference is within 90° of the radial direction. For example, a neuron whose RF is centered to the right side of the visual field along the horizontal midline has direction selectivity within 90° of the rightward direction, directions in between upward and downward are possible. At the extremes *σ_d_* = 0° would imply that population direction selectivity would strictly follow a radial pattern and *σ_d_* = 360° would imply uniform random direction selectivity across MT.

#### MT speed selectivity

In the default model we sampled speed preferences from a uniform distribution spanning the dynamic range of speeds present in the optic flow inputs:

(4) $$s^{*}∼U\left[\min\vec{s},\max\vec{s}\right],$$

where $\vec{s}$ represents the optical speeds of the 300 input motion vectors, computed as:

(5) $$\vec{s}=\sqrt{{\left(\vec{\dot{x}}\right)}^{2}+{\left(\vec{\dot{y}}\right)}^{2}}.$$

#### MT unit net input

The input signal to each MT unit depends jointly on the distance of the motion from the RF center *I_c_*, the similarity between the preferred and optic flow directions *I_v_*, and the similarity between the preferred and optic flow speed *I_s_*:

(6) $$I_{MT}=\frac{1}{T}\sum_{j=1}^{T}I_{c,j}I_{v,j}I_{s,j}.$$

That is, each MT cell averages over the set of distance-weighted direction and speed inputs that it receives. We assumed that each unit had a Gaussian-shaped RF: sensitivity to optic flow is greatest nearby the RF center and declines with distance, all other factors remaining equal. The distance between the 300 optic flow vectors and each MT unit’s RF center is computed as:

(7) $${\vec{I}}_{c}=\exp\left(-\frac{\left({\left(\vec{\dot{x}}-r_{x}\right)}^{2}+{\left(\vec{\dot{y}}-r_{y}\right)}^{2}\right)}{2\sigma_{r}^{2}}\right),$$

where *σ_r_* defines to the effective RF radius, a circle of radius *σ_r_* contains ≈95*%* of the RF area. We set *σ_r_* = 6°, which compares favorably with data on MT RF size (Deangelis and Uka, 2003).

The direction input depends on the difference between the cell’s preferred angle and those present within the RF. Equality between the preferred and optic flow angles garners the highest input and the signal decreases as the mismatch grows according to a Gaussian function:

(8) $${\vec{\Delta }}_{v}=\text{atan2}\left(\vec{\dot{y}},\vec{\dot{x}}\right)-d^{*}$$

(9) $${\vec{I}}_{v}=\exp\left(-\frac{{\vec{\Delta_{v}}}^{2}}{2\sigma_{v}^{2}}\right).$$

In Equation 9, *σ_v_* = 10° represents the sensitivity to optic flow directions that deviate from the preferred direction.

Each MT unit demonstrates similar Gaussian tuning to differences between optic flow and preferred speeds:

(10) $${\vec{\Delta }}_{s}=\sqrt{{\vec{\dot{x}}}^{2}+{\vec{\dot{y}}}^{2}}-s^{*}$$

(11) $${\vec{I}}_{s}=\exp\left(-\frac{{\vec{\Delta_{s}}}^{2}}{2\sigma_{s}^{2}}\right).$$

In Equation 11, *σ_s_* = 0.5°/s represents the sensitivity to optic flow directions that deviate from the preferred speed.

#### MT unit activation

Each MT unit *m* integrates its input over time as a leaky integrator

(12) $$\frac{dm}{dt}=-\alpha_{MT}m+\left(\beta_{MT}-m\right)I_{MT},$$

where *α_MT_* = 0.1 represents the cell’s passive decay rate and *β_MT_* = 2.5 represents the upper bound on each unit’s activation.

### Model area MSTd

#### MSTd unit placement

To investigate how the placement of MSTd RFs influences heading estimates, we distributed *N_MST_* = 169 MSTd units randomly across 2D heading space with varying amounts of center-peripheral bias. We determined the position of each MSTd RF in polar coordinates (*R*, Θ), centered on the middle of the visual field $\vec{c}$. The angles (Θ) spanned 0–360° in steps of 1/*N_MST_*, while each radius (*R*) was randomly sampled from a uniform distribution and transformed by a γ function:

(13) $$x_{p}∼U\left[0,\sqrt{c_{x}^{2}+c_{y}^{2}}\right]$$

(14) $$R=x_{p}^{\gamma }.$$

The *γ* value controls the center-peripheral bias in the heading representation: *γ* < 1 produces peripheral bias, *γ* > 1 produces central bias, and *γ* = 1 produces no bias.

#### MSTd net input

Each MSTd unit receives input from MT units tuned to directions that are locally compatible with the radial optic flow field that radiates from its preferred heading direction. For example, an MSTd unit tuned to a central heading would receive input from MT cells tuned to leftward motion on the left side of the visual field and from those tuned to rightward motion on the right side of the visual field. We define the 2D position of the heading direction in the visual field as ${\vec{h}}^{*}$. The normalized vector that points in the appropriate radial direction for ${\vec{h}}^{*}$ at the position of MT unit *i*’s RF $\left({\vec{r}}_{i}\right)$ is:

(15) $${\vec{u}}_{i}=\frac{{\vec{r}}_{i}-{\vec{h}}^{*}}{\sqrt{{\left(r_{x,i}-h_{x}\right)}^{2}+{\left(r_{y,i}-h_{y}\right)}^{2}}}.$$

We subtract the preferred direction $d_{i}^{*}$ of each MT unit *i* with the local direction ${\vec{u}}_{i}$ that is appropriate for heading ${\vec{h}}^{*}$ to determine their similarity. Non-exact matches between each pair of vectors are weighted according to the cosine tuning function:

(16) $$U_{i}=\max\left(2\cos{\left(\text{atan2}(u_{y,i},u_{x,i})-d_{i}^{*}\right)}^{q}-1,0\right).$$

The power *q *≥* *1 in Equation 16 makes each MSTd unit less selective to non-exact matches, which in our testing improved the overall accuracy of heading estimates compared with a unit exponent. We set *q *=* *2 in the default model and note that the equation as written redistributes the cosine values from the interval [−1,1] to [0,1] for even powers. Multiplication by 2 and subtraction by 1 restores the original range. The max(·,0) operation represents half-wave rectification, which sets values below 0–0. Note that the cosine tuning is mathematically equivalent to a dot product between the MT direction and MSTd radial pattern template vectors that is used in some template models (Warren and Saunders, 1995; Layton et al., 2012; Perrone, 2012).

Each local pattern match signal *U_i_* is weighted by the activation of MT unit $\left(m_{i}\right)$ at the same position and by the inverse distance between the MT RF center ${\vec{r}}_{i}$ and the heading direction ${\vec{h}}^{*}$, both of which are common computations in template models (Warren and Saunders, 1995; Royden, 2002; Layton et al., 2012).

(17) $${\tilde{I}}_{MST,i}=U_{i}\frac{m_{i}}{\sqrt{2\pi \sigma_{MST}^{2}}}\exp\left(-\frac{{\left(h_{x}-r_{x,i}\right)}^{2}+{\left(h_{y}-r_{y,i}\right)}^{2}}{2\sigma_{MST}^{2}}\right).$$

The parameter *σ_MST_* controls the effective extent of the MSTd RF. In the default model, we set *σ_MST_* = 50°, which corresponds roughly to a large 100° RF size in agreement with *in vivo* MSTd cell properties (Tanaka et al., 1986).

The net input signal that each MSTd unit receives is computed by Equation 17, averaged over the number of MT inputs:

(18) $$I_{MST}=\frac{1}{N_{MT}}\Sigma_{j=1}^{N_{MT}}{\tilde{I}}_{MST,j}.$$

#### MSTd activation

Each MSTd unit *M* integrates its input over time as a leaky integrator

(19) $$\frac{dM}{dt}=-\alpha_{MST}M+\left(\beta_{MST}-M\right)I_{MST},$$

where *α_MST_* = 0.1 represents the cell’s passive decay rate and *β_MST_* = 2.5 represents the upper bound on each unit’s activation.

### Simulation experiments

We performed three main simulation experiments on model area MSTd involving the distribution of units along the central-peripheral axis, RF size, and direction selectivity. We performed two additional experiments on model area MT focusing on direction selectivity and speed selectivity. Table 4 enumerates specified parameter values used in each experiment.

**Table 4:** Parameters varied from default model in simulation experiments

Experiment	Description	Parameter	Values
MSTd heading	left-peripheral bias in heading representation	*γ*	[0.1,0.2,0.5,1,2,5,10]
MSTd RF size	SD of Gaussian RF	*σ_MSTd_*	[11.3°, 21.8°, 38.7°, 50.2°, 58.0°, 63.4°] ([12.8, 25.6, 51.2, 76.8, 102.4, 128] pixels)
MSTd direction	Degree of intolerance to MT inputs that mismatch the preferred radial pattern	q	[1,2,4,6,8]
MT direction	maximum extent of random deviation between the radial direction and each MT preferred direction	*σ_d_*	[0°, 60°, 120°, 180°, 240°, 300°, 360°]
MT speed	Baseline beta distribution shape used to sample speed preferences of each MT unit	*k_s_*	4
MT speed + RF	Intercept of linear regression fit that determines RF size from eccentricity	*β_ecc_*_,0_	0.19° (0.42 pixels)
MT speed + RF	Slope of linear regression fit that determines RF size from eccentricity	*β_ecc_*_,1_	[0.27, 0.54, 0.81, 1.07]° ([0.3, 0.6, 0.9, 1.2]) pixels

### Experiment: MSTd central-peripheral heading tuning

We varied the model parameter *γ* (Eq. 14) to affect the distribution of heading tuning along the center-peripheral axis: *γ* < 1 produces peripheral bias, *γ* > 1 produces central bias, and *γ* = 1 produces no bias.

### Experiment: MSTd RF size

We varied the *σ_MSTd_* SD parameter that controls the extent of the Gaussian MSTd RF (Eq. 17).

### Experiment: MSTd direction selectivity

We varied the exponent parameter *q* in Equation 16 that controls the sensitivity of each MSTd cell to MT inputs that deviate from the radial directions.

### Experiment: MT direction selectivity

We varied the *σ_d_* SD parameter that controls the maximum random deviation of each MT preferred direction from a radial pattern consistent with a central heading (Eq. 3).

### Experiment: MT speed tuning

We implemented several alternatives to the uniform random model of MT speed tuning. We considered a model that discards speed information and bases heading estimates on direction alone. We implemented this model by omitting *I_s_* from Equation 6.

Additionally, we implemented a probabilistic model wherein MT cells are more likely to demonstrate faster speed tuning at greater eccentricities (Maunsell and Van Essen, 1983a; Mikami et al., 1986). We parameterized the range of MT speed sensitivity around the dynamic range of the optic flow input. Each cell’s preferred speed s* obeys:

(20) $$s^{*}=x_{s}(\max\vec{s}-\min\vec{s})+\min\vec{s},$$

where $\vec{s}$ represents the optical speeds of the 300 input motion vectors (Eq. 5). In Equation 20, *x_s_* represents a random variate between 0 and 1 sampled from the beta distribution:

(21) $$x_{s}∼\text{Beta}\left[a_{s},b_{s}\right].$$

**Figure 4.**
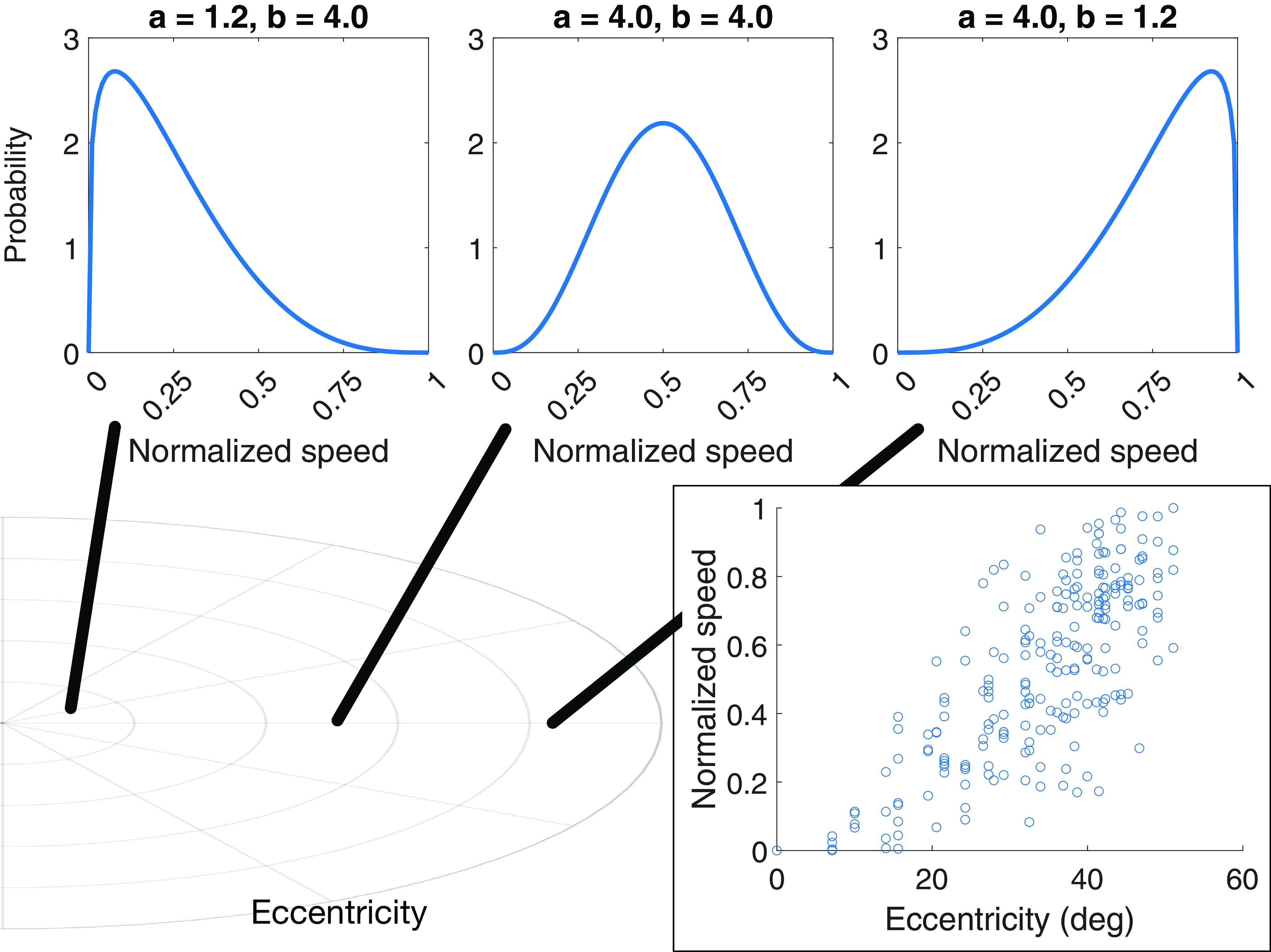
Methodology used to scale speed preference with eccentricity in MT speed tuning experiment. The eccentricity of a MT unit’s RF (bottom left) is used to parameterize the beta distribution that is sampled to determine the preferred speed (top). For example, the preferred speed of a foveal MT unit is drawn from beta distribution with most of the density concentrated around slow speeds (top-left plot). The bottom-right scatter plot shows the preferred speeds in the simulated population normalized to range of the optic flow speeds present in the stimulus.

As Figure 4, top panel, illustrates, the bell-shaped beta density is centered when the shape parameters *a_s_* and *b_s_* are equal. When *b_s_* remains fixed and *a_s_* < *b_s_*, the mean shifts left of center and the density concentrates around smaller x values (i.e., normalized speeds). Conversely, the mean shifts right of center when *a_s_* remains fixed and *a_s_* > *b_s_*. Notice how the beta distribution gracefully handles the boundary conditions as the density concentrates nearby the minimum and maximum normalized speed values.

We tailored the shape parameters *a_s_* and *b_s_* around the eccentricity of each MT neuron’s RF: the beta density spanned a continuum between being concentrated around small normalized speeds for cells at small eccentricities and around large normalized speeds for cells at large eccentricities (Fig. 4). To compute *a_s_* and *b_s_*, we calculated the eccentricity of each MT unit *i* as the normalized Euclidean distance *E_norm_*_,_*_i_* relative to the center of the visual field:

(22) $$E_{dist,i}=\sqrt{{\left(r_{x,i}-c_{x}\right)}^{2}+{\left(r_{y,i}-c_{y}\right)}^{2}}$$

(23) $$E_{norm,i}=\frac{E_{dist,i}}{\sqrt{c_{x}^{2}+c_{y}^{2}}}.$$

For MT cells with RFs that are positioned closer than halfway to the center of visual field, we configured the shape parameters to place the mean of beta density on the left side of the support:

(24) $$a_{s}=k_{s}\left(\frac{E_{norm}}{1-E_{norm}}\right)$$

(25) $$b_{s}=k_{s}.$$

For MT cells with RFs that are positioned halfway or farther away from the center of visual field, we configured the shape parameters to place the mean of β density on the right side of the support:

(26) $$a_{s}=k_{s}$$

(27) $$b_{s}=k_{s}\left(\frac{1}{E_{norm}}-1\right).$$

We fixed the baseline beta shape *k_s_* = 4 after simulations revealed that values 1–10 did not impact the accuracy of model heading estimates.

Finally, we considered a speed tuning model that combines the eccentricity dependent speed scaling (Fig. 4) with RF size scaling. We adapted the linear regression model that relates MT RF size and eccentricity scaling from Tanaka et al. (1986). The regression model has the following form:

(28) $$y_{size}=\beta_{ecc,0}+\beta_{ecc,1}x_{ecc},$$

where *x_ecc_* is the RF eccentricity in degrees, *y_size_* is the square root of the MT RF area, *β_ecc_*_,0_ is the intercept, and *β_ecc_*_,1_ is the slope. We re-parameterized the fitted coefficients from the published values to *β_ecc_*_,0_ = 0.19° and *β_ecc_*_,1_ = 0.27° since our MT model RF (*σ_r_*; Eq. 7) is expressed with respect to RF radius rather than area.

### Simulation protocol

We computed the mean and variance of heading estimates over 50 runs of each simulation experiment. The optic flow stimuli remained the same across runs and variability in estimates reflects the random sampling of model tuning parameters on each run. We repeated this process 10 times for the noise stimuli, with optic flow stimuli generated anew each time.

We used population vector decoding to estimate heading from the MSTd activation on each frame (Georgopoulos et al., 1986):

(29) $$\tilde{h}=\frac{1}{\sum_{i=1}^{N_{MST}}M_{j}}\sum_{j=1}^{N_{MST}}h_{x,j}^{*}M_{j}.$$

We focused on decoding the *x* coordinate of the heading direction given that the y coordinate remained constant across our stimuli. We computed a single horizontal heading estimate over each 60-frame video using an exponential moving average with parameter *λ*:

(30) $$h_{est,t}=\lambda {\tilde{h}}_{t}+\left(1-\lambda \right)h_{est,t-1},$$

where *t* represents the current time step. We set *λ* = 0.25 to dampen instantaneous fluctuations in heading estimates using the recent history.

### Software accessibility

We implemented the model and performed simulations in MATLAB. The model code is available on GitHub https://github.com/owlayton/Heading-MT-MSTd-Physiology-Model.

## Results

We organized our computational investigation into two parts. First, we focused on the impact of MSTd tuning on heading estimates (central-peripheral representation of heading, direction tuning, RF size). Second, we evaluated how MT tuning characteristics (direction tuning, speed tuning, and RF size) influenced heading signals in two qualitatively distinct MSTd models.

### Effect of Central-peripheral heading tuning in MSTd

**Figure 5.**
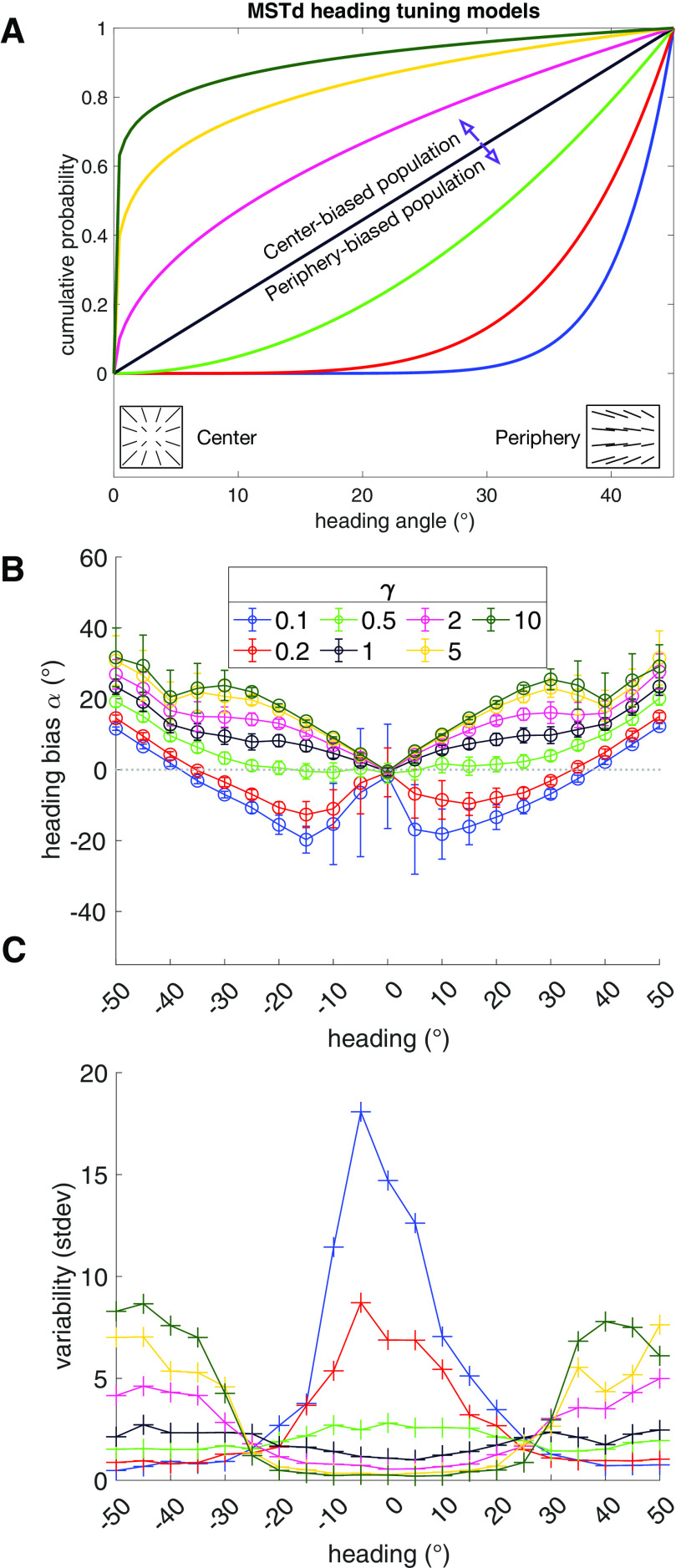
Simulations showing the effect of the heading representation of MSTd on estimates. ***A***, The cumulative probability of sampling each MSTd unit’s preferred heading according to different MSTd models. The distribution of heading selectivity across the center-peripheral axis depends on each model’s *γ* value. Only positive (rightward) headings are depicted, but the model samples headings from all 2D headings within the field of view. ***B***, Heading bias produced by simulating each model with self-motion along central-peripheral azimuthal axis within ±45° of center. Negative (positive) biases correspond to heading errors toward the periphery (center). Negative (positive) heading values correspond to self-motion to the left (right) of the straight-ahead direction, respectively. Each point indicates the mean over 50 model runs and error bars show ±1 SD. ***C***, Variability (in SDs) obtained in the simulations plotted in ***B***.

We constructed seven different models of MSTd to investigate how the representation of heading could impact the accuracy and precision of estimates. Each model is distinguished by its value of *γ*, the parameter that controls the extent to which the population heading preferences are biased along the central-peripheral axis (Fig. 5*A*): *γ* < 1 results in an overrepresentation of peripheral headings, *γ* > 1 results in an overrepresentation of central headings, and *γ* = 1 results in a uniform distribution of headings. Each MSTd model receives input from MT units, whose RFs are arranged in a regular grid instead of being randomly distributed. We made this decision to reduce internal model variability across simulations for factors unrelated to the main manipulation (*γ*). However, simulations revealed the two methods of placing MT unit RFs did not differ in the average accuracy or the precision of the heading estimates (Extended Data Fig. 5-1).

10.1523/ENEURO.0307-21.2021.f5-1 Extended Data Figure 5-1 Effect of MT cell placement. Our goal for the simulations depicted in Figure 5 was to focus on the influence of MSTd tuning on heading estimation while maintaining a consistent model MT. Toward this end, we distributed MT units in a 2D rectilinear grid; positioning MT RFs randomly, as we do in MSTd, might contribute error and variability to heading estimates unrelated to MSTd. Given that an even distribution of MT RFs across visuotopic space may not be a plausible assumption, we repeated the simulations whose results are shown in Figure 5 wherein MT RFs were placed randomly for two of the MSTd tuning models. As this figure shows, there is no meaningful difference in mean heading errors and variability when placing MT units randomly and on a regular 2D grid. Download Figure 5-1, EPS file.

Figure 5*B* shows the heading bias produced by each MSTd model for the 3D dot cloud optic flow stimuli. Heading bias is defined as the error in heading estimates, toward (positive) or away from (negative) the center (Fig. 1*D*). As expected, models with an overrepresentation of central headings (*γ* > 1) produced the lowest error when estimating central headings, while models with an overrepresentation of peripheral headings produced the lowest error when estimating peripheral headings (*γ* < 1). It is noteworthy that all models, even those that overrepresent peripheral headings (*γ* < 1), did not accurately estimate headings in the far periphery. This occurred because the FoE and surrounding motion may not appear within the 90° field of view. Motion nearby the FoE provides important information about heading (Crowell and Banks, 1996) and in its absence, MSTd heading detectors cannot estimate heading as accurately. The error is in the direction of the available motion vectors, resulting in an underestimation of heading (i.e., center bias).

Simulations reveal that a model with a weak-to-moderate overrepresentation of peripheral headings (*γ* = 0.5) garnered the best overall accuracy [i.e., least absolute bias; mean absolute error (MAE) = 5.7°] across all of the conditions. This was the only model that garnered constant, nearly zero bias for central headings ≤±20°. The other MSTd models produced two qualitatively distinct patterns of bias. Models with a more extreme peripheral heading overrepresentation (*γ* < 0.5) exhibited peripheral bias in their estimates of the central headings, approximately within ±35°. Models with an overrepresentation of central headings (*γ* > 1) demonstrated consistent center bias that grew with heading eccentricity. The model with uniform heading sensitivity (*γ* = 1) behaved similarly to those with an overrepresentation of central headings.

Figure 5*C* plots the variability in heading estimates obtained over the 50 runs of each model. For MSTd models with an overrepresentation of central headings, variability was small in the center and large in the periphery. The opposite was true for models that had an overrepresentation of peripheral headings: large variability for central heading and small variability for peripheral headings. Notably, the model that garnered the best overall accuracy (*γ* = 0.5) garnered the lowest overall mean variability (1.7 SDs). It also produced the most consistent variability across headings. Together, these results suggest that a MSTd model with the weak-to-moderate overrepresentation of peripheral headings yields the most accurate and precise heading estimates.

**Figure 6.**
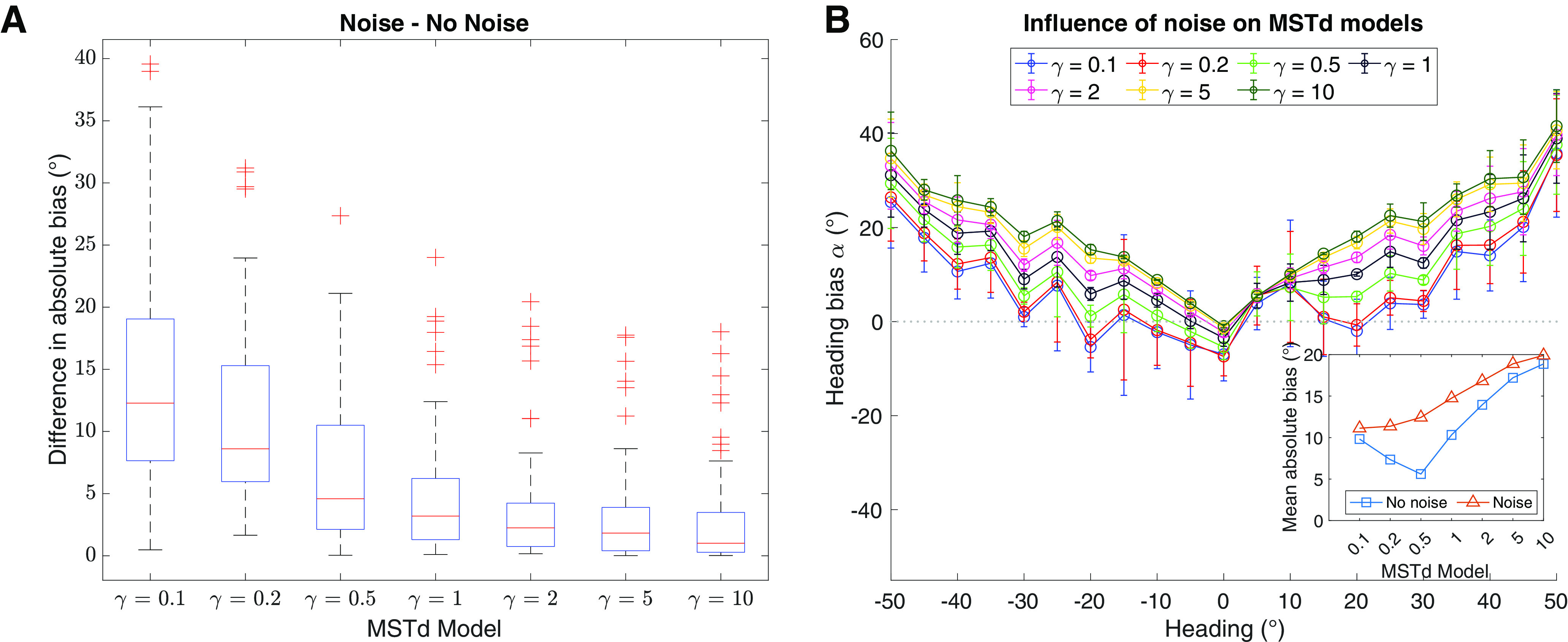
Simulations of optic flow containing noise using MSTd models with different heading representations. ***A***, Box plot showing the absolute difference between the no noise condition and the noise stimuli that had 21 different headings and three noise levels (70%, 80%, or 90% noise dots). ***B***, Mean heading bias averaged across noise levels produced by each MSTd model for each heading (same plotting conventions as Fig. 5). Inset compares mean absolute heading bias between the noise (red) and no noise (blue) stimuli, averaged across individual heading directions.

Next, we examined how the estimates produced by the different MSTd models were influenced by optic flow that differs from the idealized patterns used to construct each MSTd unit’s RF. We simulated each model using stimuli wherein we replaced 70–90% of the dots belonging to the 3D dot cloud with noise uncorrelated with the self-motion direction (Fig. 2*B*). We henceforth refer to stimuli without noise as the “no noise” condition. Figure 6*A* shows how the 63 heading estimates produced each MSTd model (21 headings × three noise levels) deviated from the no noise condition. The median effect of noise in most cases was only several degrees despite the considerable differences in the heading representations across the models. While the central MSTd models (*γ* > 1) were only weakly affected by the noise, the baseline accuracy of these models is poor regardless of noise, especially compared with the peripheral models (compare Figs. 5*B* and 6*B*). For example, without noise the *γ* = 10 model exhibits 30° heading error toward the center for ±50° headings.

Noise did, however, influence MSTd models with an overrepresentation of peripheral headings (*γ* < 0.5) to a greater extent, as indicated by the much higher medians and presence of more extreme absolute differences from the no noise condition. The model with a weak-to-moderate overrepresentation of peripheral headings (*γ* = 0.5) demonstrated the biggest mean shift in the accuracy of its estimates (Fig. 6*B*, inset). Recall that this model garnered the most accurate estimates in the no noise condition. The greater influence of noise in the peripheral models increased the center bias (i.e., positive shift) for most headings (compare Figs. 5*B* and 6*B*). Despite the increased sensitivity to noise, peripheral MSTd models still produced the most accurate heading estimates (Fig. 6*B*, inset).

### Effect of MSTd RF size

MSTd neurons are well known to exhibit tremendous variation in their RF size, reaching 100° or more (Tanaka et al., 1986). We explored within the computational framework whether such large RF sizes are necessary to support accurate heading estimation. Given the qualitatively different patterns of heading bias produced by the center and peripheral MSTd models, we henceforth focus on two specific model instances: one with an overrepresentation of peripheral headings (*γ* = 0.5; henceforth “peripheral model”) and one with an overrepresentation of central headings (*γ* = 2; henceforth “central model”).

**Figure 7.**
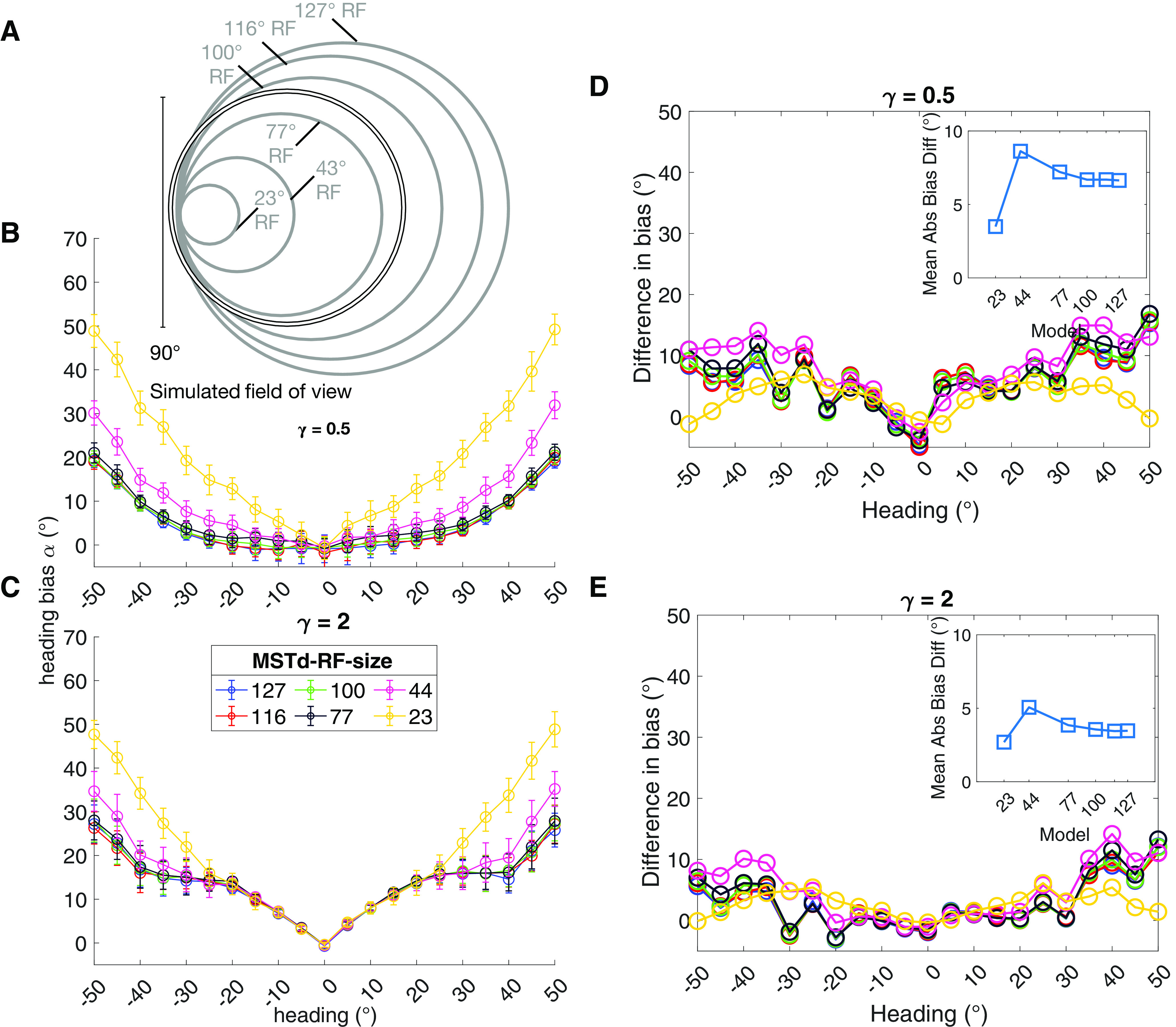
Simulations showing the effect of MSTd RF size on heading estimates. ***A***, Schematic showing the simulated MSTd RF sizes (gray circles) compared with the 90° simulated field of view (black circle). ***B***, ***C***, Heading bias obtained over 50 runs of the peripheral (***B***; *γ* = 0.5) and central (***C***; *γ* = 2) MSTd models. ***D***, ***E***, Difference in mean heading bias produced by the peripheral (***D***; *γ* = 0.5) and central (***E***; *γ* = 2) MSTd models between the noise and no noise conditions. Insets show the absolute difference in heading bias between the noise and no noise conditions averaged across headings. Positive values indicate increased bias toward the straight-ahead.

Figure 7*A* schematizes the simulated MSTd RF diameters (gray circles) that ranged from 23° to 127° relative to the 90° field of view (black circle). RFs larger than the field of view are worth considering because such units tuned to peripheral headings are capable of integrating motion at the far reaches of the opposite side of the visual field. Simulations of the peripheral model showed that RF size made little difference when estimating central headings (Fig. 7*B*). The effect of RF size was different at the periphery: increasing MSTd RF size improved heading accuracy, but the improvement plateaued once the RF size reached 77°. The small 23° units produced more than twice the bias for peripheral headings than the larger units toward the center. This suggests that integrating contextual information over a larger area helps to mitigate the uncertainty in the periphery caused by the lack of a FoE within the field of view. Figure 7*C* shows the estimates produced by the central model. Once again, it produced less accurate heading estimates overall than the peripheral model. While the pattern of bias is similar, the increasing the RF size did not improve the estimates as much.

Figure 7*D*,*E* shows the difference in bias between the noise and no noise conditions. On average, noise had a similar impact regardless of MSTd RF size. In all but the smallest RF size, there was an increase in the heading bias toward the center at peripheral headings, but for most headings the effect was weak. The peripheral MSTd model exhibited the same qualitative effect, but demonstrated increased sensitivity compared with the central model.

### Effect of MSTd direction tuning

**Figure 8.**
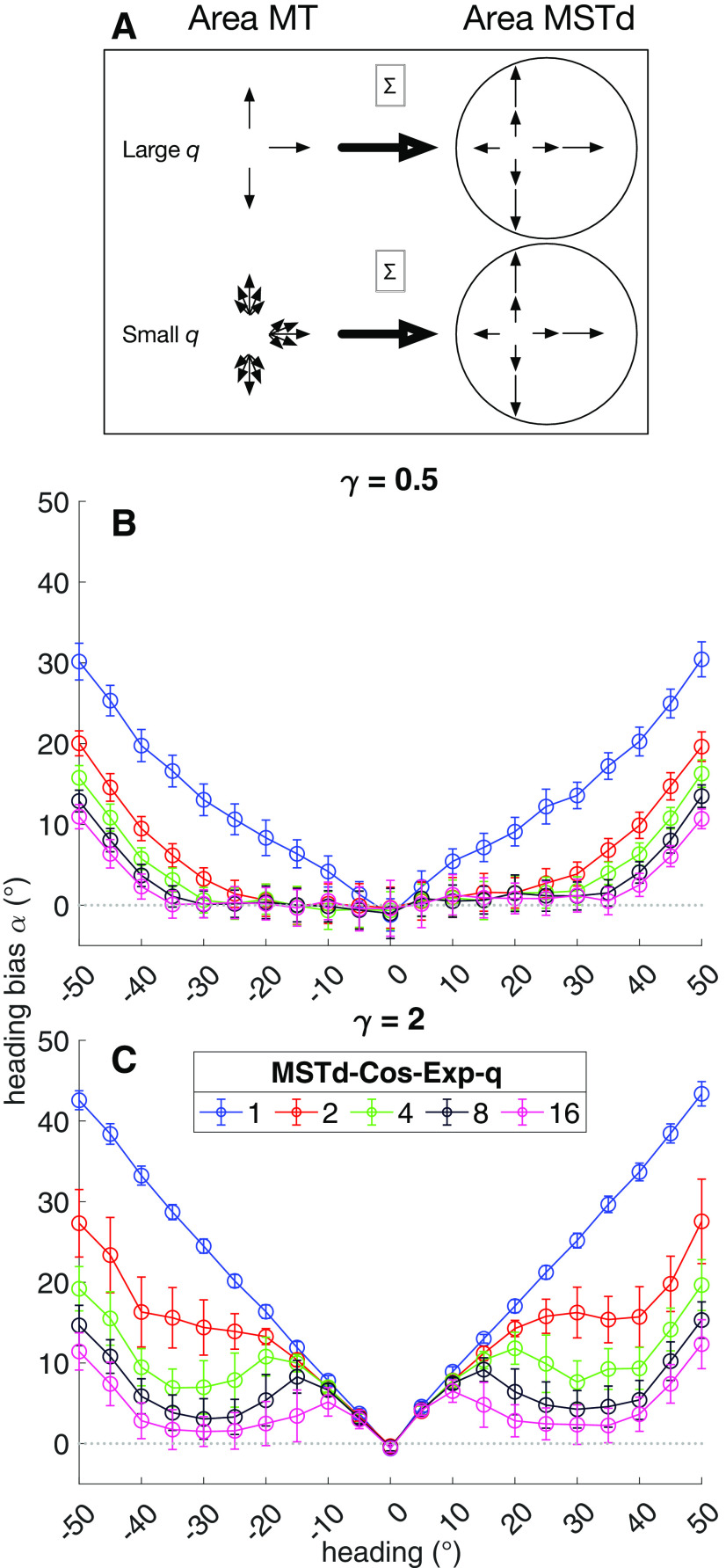
Simulations focusing on direction selectivity in MSTd. ***A***, We varied model parameter *q* that controlled the range of directions each MSTd unit integrated that deviated from the preferred radial pattern. Large *q* values increased the selectivity and in the extreme case, MSTd units would only integrate directions that exactly match the preferred radial pattern. Smaller *q* values increased the range of integrated directions, centered around each preferred radial direction. ***B***, Mean bias for each heading direction produced by the peripheral MSTd model configured with different *q* values. ***C***, Same as ***B*** except for the central MSTd model. Same plotting conventions as Figure 5.

A key characteristic of template models of MSTd is that RFs are constructed from idealized patterns of radial optic flow (Fig. 1*A*,*C*). Some template models make the strong assumption that each MSTd unit integrates only the set of MT directions that form the specific global pattern (Fig. 8*A*, top; Royden, 2002; Layton and Fajen, 2016b), while others integrate at every visuotopic position a range of directional inputs centered on the direction consistent with the radial pattern (Fig. 8*A*, bottom; Perrone, 1992; Raudies and Neumann, 2010). Before turning our focus to the impact of MT tuning on heading signals, we examined the extent to which the selectivity of each MSTd unit to directional inputs influences heading estimates.

We manipulated the selectivity of MSTd directional inputs that deviate from each radial direction via the commonly used rectified cosine tuning function. This function weights the difference between the preferred radial direction and preferred directions of the MT units that send input (Eq. 16). The function ensures that the MSTd unit still responds maximally to the preferred heading because discrepant directions are down-weighted by the cosine function. To control the range of directional inputs that may activate the MSTd unit, we raised the cosine to the power *q* (Fig. 8*A*).

Figure 8*B* shows the heading bias produced by the peripheral model. Increasing the directional selectivity substantially improved the accuracy across all non-zero headings. The most pronounced increase in accuracy occurred from *q *=* *1 and *q *=* *2 and higher exponents further improved the estimates in the periphery. The highest exponent values tests garnered accurate heading estimates for approximately the central 70° heading directions. The central model yielded much less accurate heading estimates overall (Fig. 8*C*). However, the effect of directional selectivity is similar to that in the peripheral model: larger powers reduced heading bias, albeit to a lesser extent.

In both models, simulations involving powers >3 had the undesirable effect of making MSTd units so narrowly tuned to direction that occasionally some optic flow inputs would not activate any of the simulated MSTd units. This occurred because we simulated a limited number of MSTd units and there was no guarantee that MSTd contained a unit tuned to the exact heading in the optic flow input. In these situations, we re-ran the model with different random initial conditions until we obtained 50 valid heading estimates. Taken together, these simulations suggest that increased directional selectivity to the preferred pattern supports accurate self-motion estimation over a wider range of headings. However, extremely narrow direction tuning has the adverse effect of making neurons too selective for patterns, even for those that deviate modestly from their preferred radial pattern of motion.

### Effect of MT direction tuning

**Figure 9.**
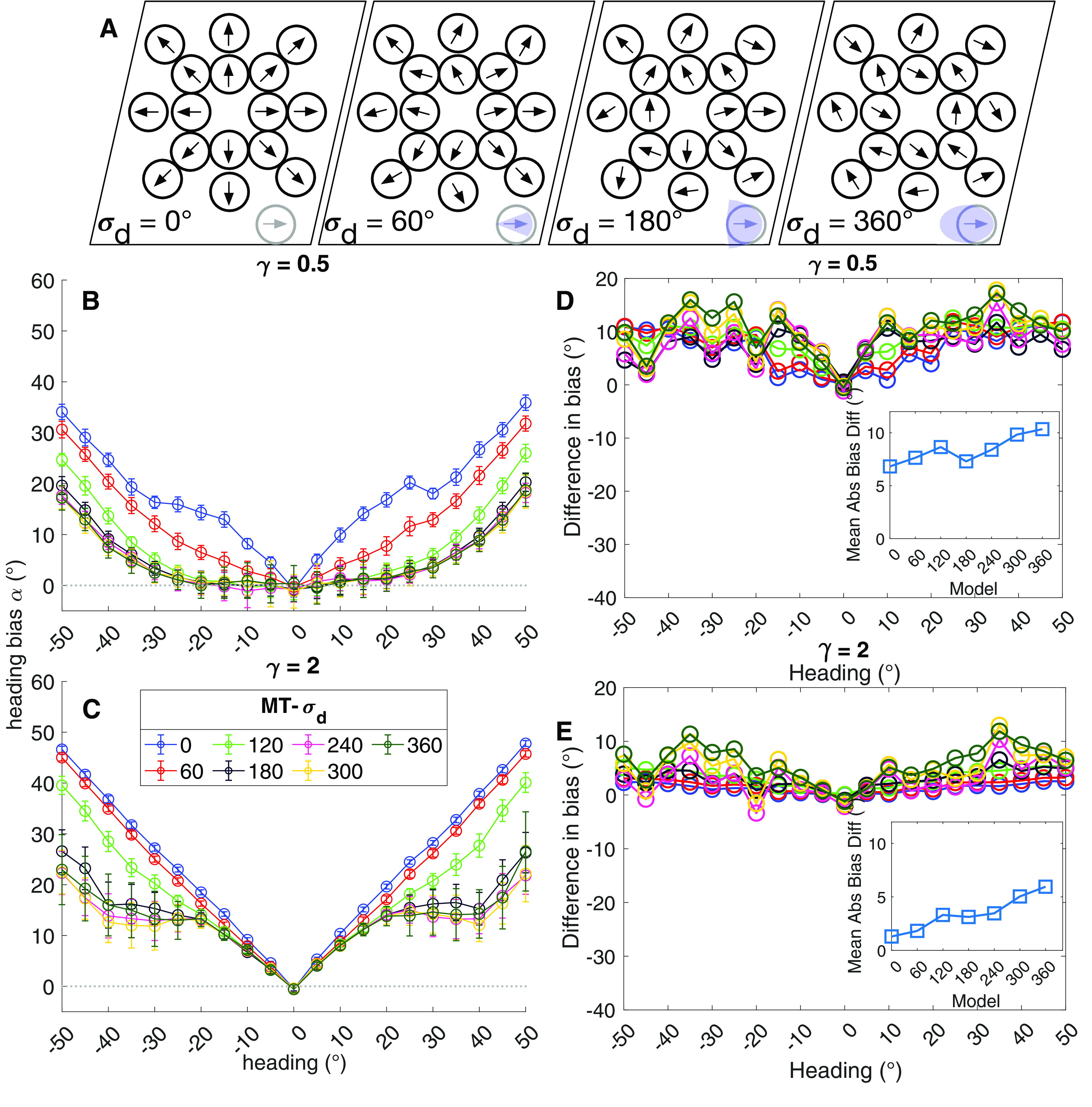
Simulations focusing on the effect of MT direction tuning on MSTd heading signals. ***A***, Schematic illustrating the influence of the parameter *σ_d_* on MT direction selectivity. Values close to 0° result in direction tuning that resembles a radial pattern across the population. Larger values define the maximum bilateral extent to which MT units may deviate from the radial direction. The *σ_d_* = 360° condition created uniform random selectivity across MT. ***B***, ***C***, Heading bias obtained over 50 runs from the peripheral (***B***; *γ* = 0.5) and central (***C***; *γ* = 2) MSTd models. Same plotting conventions as Figure 5. ***D***, ***E***, Difference in mean heading bias produced by the peripheral (***D***; *γ* = 0.5) and central (***E***; *γ* = 2) MSTd models between the noise and no noise conditions. Same plotting conventions as Figure 7.

Template models of MSTd predict that heading sensitivity largely emerges through the feedforward integration of MT signals. We explored how MT direction and speed tuning could influence heading estimate downstream in MSTd. We parameterized model MT direction tuning around the known population bias toward a global radial pattern (Albright, 1989). For example, MT units with RFs on the right side of the visual field are more likely to be tuned for rightward than leftward motion (Fig. 9*A*, leftmost panel).

We tested how anchoring MT direction tuning to a radial pattern impacts model heading estimates. Figure 9*A* schematically depicts a continuum of constraints on the model direction tuning, ranging from strictly radial (left; *σ_d_* = 0°) to uniform random (right; *σ_d_* = 360°). Strongly enforcing the radial direction constraint (*σ_d_* ≈ 0°) resulted in substantial heading bias toward the center, giving rise to V-shaped bias curves in both peripheral (Fig. 9*B*) and central (Fig. 9*C*) MSTd models. Constraining the direction tuning within ±90° of the radial direction (*σ* ≤ 180°) improved the accuracy produced by both MSTd models in the periphery. Relaxing the tuning constraint beyond ±90° did not further improve the accuracy. This coincides with the fact that the local direction of the optic flow cannot differ >90° in the optic flow stimuli that we used wherein heading varied along the horizon.

Estimates produced by the peripheral MSTd model were more greatly affected by noise than those produced by the center model (Fig. 9*D*,*E*). In both models, the direction of the bias was toward the center. Taken together, this indicates that noise shifted MSTd activation toward neurons tuned to central headings to a greater extent than in the no noise condition, despite the more limited resources dedicated toward representing central headings. The center bias grew in magnitude in both models when MT tuning deviated from the radial direction (Fig. 9*D*,*E*, insets). This occurred because random direction selectivity in MT is uncorrelated with radial motion. Therefore, a greater proportion of the MT units signal noise in this case rather than the motion because of observer self-motion, which decreases sensitivity to the global pattern. It is noteworthy that the central MSTd model was only affected by noise when MT direction tuning was more randomly distributed (Fig. 9*E*). This suggests that increased sensitivity to central headings compensates for the less selective direction signals in MT.

It is important to emphasize that despite its increased sensitivity to noise, the peripheral model still generally produces at least as accurate estimates as the central model. This is because the peripheral model demonstrates ≈5° more mean bias than the central model (compare Fig. 9*D*,*E*, insets), yet for many headings the baseline accuracy of the peripheral model is at least ≈5° more accurate, sometimes considerably more so (compare Fig. 9*B*,*C*).

### Effect of MT speed tuning

**Figure 10.**
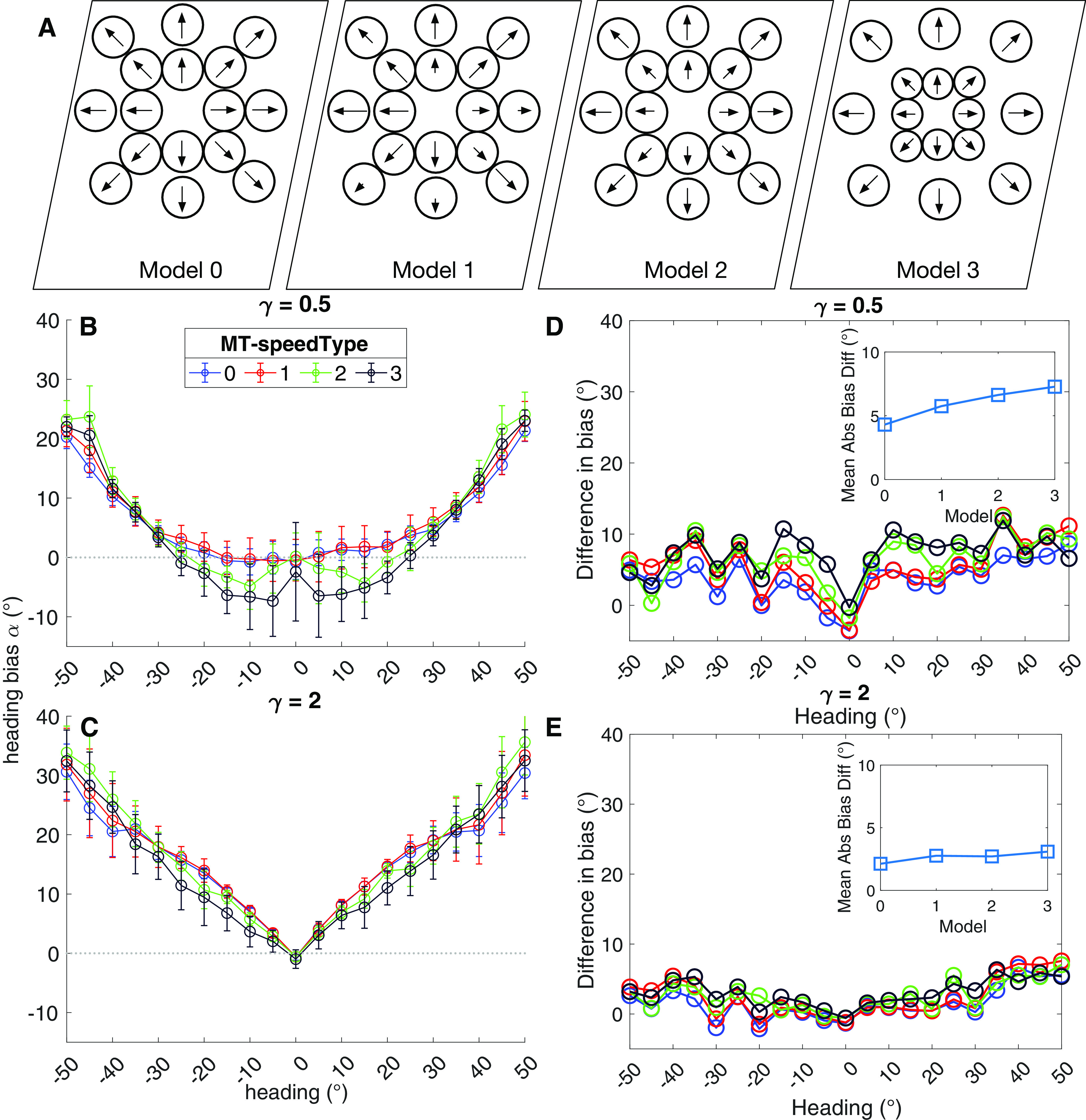
Simulations focusing on the effect of MT speed tuning on MSTd heading signals. ***A***, Schematic depiction of the four MT speed tuning models considered: (0) direction only, (1) uniform random, (2) likelihood of faster preference increases with eccentricity, (3) likelihood of faster preference and RF size increase jointly with eccentricity. ***B***, ***C***, Heading bias obtained over 50 runs from the peripheral (***B***; *γ* = 0.5) and central (***C***; *γ* = 2) MSTd models. Same plotting conventions as Figure 5. ***D***, ***E***, Difference in mean heading bias produced by the peripheral (***D***; *γ* = 0.5) and central (***E***; *γ* = 2) MSTd models between the noise and no noise conditions. Same plotting conventions as Figure 7.

We considered four different models of MT speed tuning (Fig. 10*A*). In model 1, we sampled MT speed preferences from a uniform random distribution. In model 2, the probability that MT units were tuned to faster speeds increased with the eccentricity of their RFs (Maunsell and Van Essen, 1983b). Model three combines model 2 with increasing RF size as a function of eccentricity (Tanaka et al., 1986; Raiguel et al., 1995). Finally, we considered a model wherein heading signals were determined on the basis of direction alone, ignoring speed (model 0).

Figure 10*B* shows the heading estimates produced by the peripheral MSTd model combined with the four MT speed models. Only the direction-only (model 0) and uniform random (model 1) models accurately estimated heading for central headings. Model 2 with its increased likelihood of faster speed tuning at greater eccentricities produced peripheral bias for non-zero central headings. Also increasing the RF size with eccentricity (model 3) amplified this bias. All four MT speed models demonstrated similar bias toward the center for peripheral headings. In the case of the center MSTd model (Fig. 10*C*), none of the speed models appreciably impacted heading estimates. Finally, Figure 10*D*,*E* reveals that the center MSTd model was not substantially impacted by noise with any of the MT speed tuning models. The peripheral MSTd model exhibited increased sensitivity to noise, particularly in the models that scaled speed tuning with eccentricity (models 2 and 3).

## Discussion

The present simulation study quantifies how physiological tuning characteristics in MT and MSTd influence heading signals within the template model computational framework. Physiological parameters in both areas exerted large, diverse effects on heading estimation. To encourage broad applicability of our results, we did not simulate any one specific model; rather, we simulated a combination of physiological constraints and core computations that broadly encapsulate mechanistic models of MT and MSTd.

### The impact of MSTd physiology on heading estimates

Our findings broadly support the notion that tuning properties in MSTd optimize the accuracy of heading estimation subject to neurophysiological constraints. In particular, we found that an overrepresentation of peripheral headings in model MSTd yielded the most accurate and precise heading estimates of the models tested. The model demonstrated sensitivity to noise, however, despite the noise, estimates remained more accurate than MSTd models with an overrepresentation of central headings. Physiological studies have also reported peripheral bias in the heading tuning of MSTd neurons (Lappe et al., 1996; Gu et al., 2006). Such a model may perform well because the steepest portion of tuning curves across the population may capture a range of headings (Gu et al., 2010). We found that the peripheral bias in the heading representation also supported heading accuracy in the far periphery, despite the uncertainty created by the FoE falling near or outside the edge of the field of view. Note, however, that MSTd models with more extreme peripheral bias did not perform as well. Available neural data appears to support a weak-to-moderate peripheral bias consistent with the best performing model: the largest number of MSTd neurons possess tuning to eccentric headings but not to those in the far periphery (Duffy and Wurtz, 1995; Gu et al., 2006).

### Large RFs and noise tolerance

The presence of MSTd neurons with large RF sizes spanning much of the visual field has long been noted (Tanaka et al., 1986; Duffy and Wurtz, 1991). Of the MSTd RF sizes that we tested, 77° and greater similarly supported the accuracy of heading estimates. The main advantage of such large RF units in our simulations was improved accuracy for peripheral heading estimates; units with RF sizes <50° did not demonstrate much difference when estimating central headings. Remarkably, 70–90% noise had a small average effect (5–10° mean difference in bias), even in MSTd models with small RF units. This agrees with the accuracy of human heading perception under similarly low signal-to-noise ratio conditions (Van Den Berg, 1992). It also supports what other computational studies have noted, that the computational mechanism by which MSTd neurons integrate motion signals according to radial templates can be robust under diverse conditions, even when the optic flow deviates from the preferred template patterns (Perrone, 1992; Royden, 2002; Browning et al., 2009; Layton and Fajen, 2016a).

### Human perception of peripheral headings

**Figure 11.**
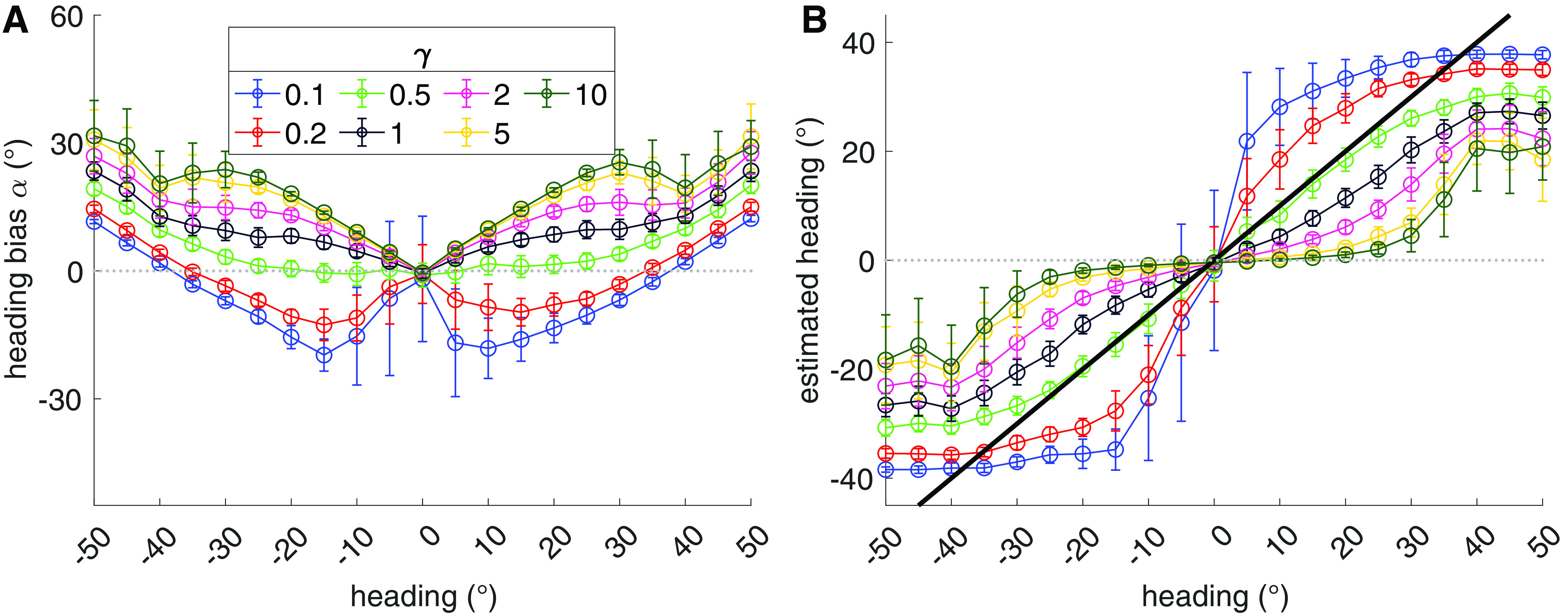
Simulation results from Figure 5*B* expressed with respect to bias (***A***) and raw heading estimates (***B***). Thick black curve in ***B*** indicates unity line. Same plotting conventions as Figure 5.

While the accuracy of humans heading judgments decreases with eccentricity, judgments plateau toward the periphery. This forms an approximately S-shaped (i.e., sigmoidal) curve (D’Avossa and Kersten, 1996, see their see Fig. 1; Sun et al., 2020, see their see Fig. 2). Although this may seem at odds with the general pattern presented here whereby bias increases with eccentricity (Fig. 5*B*), it is important to emphasize that we have plotted bias rather than actual estimates (“judgments”). Figure 11 depicts the results obtained from the MSTd heading tuning simulation experiment (Fig. 5), both as heading bias (Fig. 11*A*) and raw heading estimates (Fig. 11*B*). The S-shaped curve readily emerges for each MSTd model. Sun et al. (2020) similarly show in their Figure 2*A*,*B* that human heading bias rises linearly and raw judgments follow an S-shaped curve.

Toward the periphery, however, the amount of predicted bias diverges from available human data, which generally show bias that does not exceed ≈10° (Crane, 2012; Cuturi and Macneilage, 2013). Models with extreme overrepresentations of peripheral headings (*γ* = 0.1, *γ* = 0.2) are the exception, although they produce zero crossings in the bias to either side of the straight-ahead, which does not occur in human judgments of forward headings. Figure 11*B* reveals that the large peripheral bias corresponds to estimates within 20–40° of the straight-ahead. This substantial center bias arises because the FoE is close to leaving the simulated 90° field of view and the class of template models simulated here contains no mechanism to “extrapolate” the FoE position.

We are unaware of any biologically-inspired models that have demonstrated human-like heading predictions in the periphery. Computer vision algorithms that triangulate the FoE position based on visible optic flow may yield improved heading accuracy that more closely matches human performance in this scenario (Hildreth, 1992; Raudies and Neumann, 2013). However, another mechanism might be required since triangulation ought to produce maximal bias when estimating lateral headings (±90°) and human bias reaches its maximum at ±45° (Crane, 2012). One such mechanism may involve the subpopulation of MSTd neurons tuned to planar optic flow patterns (Duffy and Wurtz, 1995). Because motion vectors appear nearly parallel during self-motion along peripheral headings, planar cells may improve sensitivity to peripheral headings. Perception of peripheral headings and its underlying neural mechanisms warrant further investigation.

### Direction of human heading bias

Psychophysical studies have long observed that humans demonstrate center bias in their heading judgments (Llewellyn, 1971; Johnston et al., 1973; Cutting et al., 1992; Warren and Saunders, 1995; Royden and Hildreth, 1996; Layton and Fajen, 2016b; Sun et al., 2020). Sun et al. (2020) have examined this phenomenon and found that center bias reaches ≈10° for ±30° headings. On the other hand, some studies of human heading perception have found the opposite pattern, bias toward the periphery (Crane, 2012; Cuturi and Macneilage, 2013).

Given how many times the bias has been reproduced under different experimental conditions, the discrepancy in direction across studies is unlikely to arise from specifics with the optic flow stimuli and viewing conditions. It is still possible, however, that the discrepancy could stem, at least in part, from methodological differences rather than a neural basis. One factor that distinguishes studies that have found peripheral bias is the use of orientation to indicate subjects’ perceived heading. Subjects controlled the orientation of either a virtual arrow (Cuturi and Macneilage, 2013) or a physical dial (Crane, 2012) to match their perceived heading direction. Perhaps subjects systematically overestimate heading when making orientation-based judgments. Crane (2012) found evidence to support this possibility with his “spoken” condition: stationary subjects sitting in darkness adjusted a physical dial to match a verbally spoken heading angle. The peripheral bias that emerged was qualitatively similar to that obtained in the visual (optic flow) condition, albeit weaker in magnitude. Including the full 360° range of headings in an experimental block of the visual condition tripled the peripheral bias compared with limiting headings to ±45° (Crane, 2012), further suggesting task-dependent effects. Cuturi and Macneilage (2013) reproduced the peripheral bias using a 2AFC staircase instead of the orientation-based paradigm, but only tested it for vestibular stimuli that subjects experienced while blindfolded. It is unclear whether optic flow would yield bias of the same direction. Indeed, Warren and Kurtz (1992) used a 2AFC procedure with optic flow stimuli and found center bias.

If the direction of bias has a neural basis, the physiological characteristics of MT and MSTd analyzed here may contribute. The MSTd model with a weak-to-moderate overrepresentation of peripheral headings (*γ* = 0.5; Fig. 5*B*) captures the linear increase in center bias that reaches ≈10° at 30° eccentricity in Sun et al. (2020; see their Fig. 2*b*). However, it does not account for the variability in human heading judgments. Figure 5*C* shows that it demonstrates flat variability that decreases slightly with eccentricity, yet the average variability in human judgments grows with eccentricity (Crane, 2012; Cuturi and Macneilage, 2013; Sun et al., 2020). Interestingly, MSTd models that overrepresent central headings do demonstrate increased average variability in the periphery. In the case of the *γ* = 5 and *γ* = 10 models, the magnitude approximately matches that of human subjects (Cuturi and Macneilage, 2013; see their Fig. 3*E*). Unfortunately, these models produce too much bias (Fig. 5*B*) to plausibly account for the human data. If the *γ* = 0.5 peripheral model that is most consistent with known physiology is actually most representative of MSTd, it is possible that the variability in human judgments arises from a source not modeled here.

MSTd models with greater sensitivity to peripheral headings (*γ* < 0.5) reproduce the ≈10° peripheral bias (Crane, 2012; Cuturi and Macneilage, 2013), at least for central headings. As noted above, the simulated models qualitatively diverge from the human data in the periphery. Interestingly, the physiologically supported tendency for MT neurons to exhibit faster speed tuning and larger RFs with eccentricity yielded peripheral bias in the peripheral MSTd model (*γ* = 0.5; Fig. 10*B*), which otherwise only produced center bias (Fig. 5*B*). This suggests that MT physiology supports peripheral heading bias when interacting with MSTd. Note that the shift from center to peripheral bias emerged only for central headings and in the physiologically supported peripheral model of MSTd, not the central model (*γ* = 2). Thus, MT tuning properties do not allow any of the MSTd models considered here to fully account for the peripheral bias produced by humans.

A pervasive assumption in our model construction and interpretation has been that tuning remains fixed, yet task demands and learning substantially influence dynamics in MT (Liu and Pack, 2017) and MSTd (Jacob and Duffy, 2015; Page and Duffy, 2018). It is possible that center and peripheral bias may arise in a single model in different contexts. For example, the visual system could dynamically increase the weight of MT neurons tuned to faster speeds if they signal valuable information that correlates with heading readouts, resulting in peripheral bias. Conversely, the weight of these MT neurons could be decreased if they do not meaningfully contribute to the readout, resulting in center bias. The brain might also favor readouts from subpopulations that less accurately encode heading to trade accuracy for rapid response time. Indeed, human heading perception depends on the temporal evolution of the optic flow field (Layton and Fajen, 2016b; Burlingham and Heeger, 2020). Future work should elucidate how dynamic and task-dependent factors influence heading perception.

## Synthesis

Reviewing Editor: Li Li, New York University Shanghai Decisions are customarily a result of the Reviewing Editor and the peer reviewers coming together and discussing their recommendations until a consensus is reached. When revisions are invited, a fact-based synthesis statement explaining their decision and outlining what is needed to prepare a revision will be listed below. The following reviewer(s) agreed to reveal their identity: qi sun, Yong Gu.

Both reviewers think the ms is interesting and promising. Nevertheless, both reviewers raised important concerns that should be addressed before the ms can be considered for publication. I list their detailed comments below.

Reviewer 1

In this manuscript, the authors used computational modeling methods to systematically examine how the neurophysiological properties of MT and MST shaped heading perception from optic flow and provided insightful findings for future computational model building. Overall, the study is nicely conducted. The results are solid and clear. The analysis is thorough. I have a few comments.

1) After carefully reading the manuscript, I found that the simulation results failed to match the existing results of psychophysics studies, though the model can predict the center bias (e.g., Sun et al. 2020) and peripheral bias (e.g., Crane et al., 2013). For example, Figures 5b and 5c show that the model with certain parameters can capture the peripheral bias. The SDs of the headings around 0 deg are larger than the peripheral headings. However, Crane (2012) and Cuturi & Macneilage (2013) revealed the small SD for the headings around 0 deg as the heading perception was peripheral bias.

2) Previous psychophysics studies also showed that the heading bias increased with the increase of heading eccentricity, but the increasing trend gradually decreased (see Sun et al., 2020; Crane, 2021; etc.). However, all figures in your manuscript did not show this trend.

3) Your discussion is short and not insightful enough. You can combine your fitting results with the existing heading perception model and provide some directions for future model building.

4) In the last paragraph of page 35, for the discussions about why there was a “discrepancy", it will be better to connect their findings with your model parameters. Or, in other words, how to modulate the model parameters to explain the discrepancy.

5) Figure 1, “c” is missing.”

Reviewer 2

n the current study, the authors tried to incorporate some interesting physiological findings of the dorsal visual pathway, in particular, the area of MSTd and MT into their computational model, and predict some behavior in heading perception as seen in the human psychophysical studies. Two key factors were incorporated: 1) over representation of peripheral headings of MSTd neurons, and 2) tendency of global motion preference for MT neurons with eccentric receptive fields. When incorporating these factors, together with some others of speed and receptive sides, the authors show the model could generate best accurate and precise heading perception. Overall, the study is nicely conducted. The results are solid and clear. The analysis is thorough. I have a few comments.

1) Although the model with gamma=0.5 produces the least bias for eccentric headings, and this is a basis for the authors to conclude how model, physiology, and behavior converge, there are a few factors that may undermine this conclusion. First, the behavior studies, as the authors pointed out, have shown contradictory results. It is so far unclear what may have caused these discrepancies. The authors proposed a few possibilities, but I don't think they are likely reasons. For example, the difference in the stimulus size is only minor (∼10 degrees), and if it is really the reason, this could be easily tested in their model, and by showing this result, would the model be more useful in terms of explaining a larger range of variability in the behavior? Second, the other parameters with gamma>0.5 also seem to capture one of the behaviors, but the authors want to exclude these possibilities, simply based on the factor that they produced larger noise in accuracy and precision. It would be very hard to know whether the brain is really using a model with smallest accuracy and precision, right? It is very likely in many cases, the brain may want to sacrifice these for other purposes, for example, fast respond.

2) From the MT simulations, it seems that using the MT physiological feature, that is, global preference for MT neurons with eccentric RF, would be able to produce the behavior, without having to incorporate the MSTd physiology, correct? What is the exact relation between the two features of the MT and MSTd neurons (see above)? Could the authors give some discussion, or investigation?
